# 4-Methyl-2-[(*E*)-phen­yl(1,2,3,4-tetra­hydro-1-naphthyl­imino)meth­yl]phenol

**DOI:** 10.1107/S1600536808030316

**Published:** 2008-09-24

**Authors:** Guang-You Zhang, Ting Yang, Bao-Wang Xu, Di-Juan Chen, Wan-Hui Wang

**Affiliations:** a School of Chemistry, Jinan University, Jinan 250022, People’s Republic of China; b Qilu Pharmaceutical Co. Ltd, Shandong, Jinan 250100, People’s Republic of China; c Graduate School of Science and Engineering, Saitama University, Saitama 338-8570, Japan

## Abstract

In the crystal structure of the title compound, C_24_H_23_NO, the phenyl ring makes dihedral angles of 81.53 (11) and 75.35 (12)°, respectively, with the methyl-substituted and the fused benzene rings. The dihedral angle between the two benzene rings is 71.10 (10)°. There is an intra­molecular O—H⋯N hydrogen bond.

## Related literature

For related structures, see: Elmali & Eleman (1998 ▶); Elmali *et al.* (1998 ▶). For general background, see: Bernaldi *et al.* (1996 ▶); Cavell *et al.* (2002 ▶); Desimani *et al.* (1995 ▶); Jacobsen *et al.* (1997 ▶); Kureshy *et al.* (1996 ▶); Nakayama *et al.* (2004 ▶); Takenaka *et al.* (2002 ▶); Varlamov *et al.* (2003 ▶).
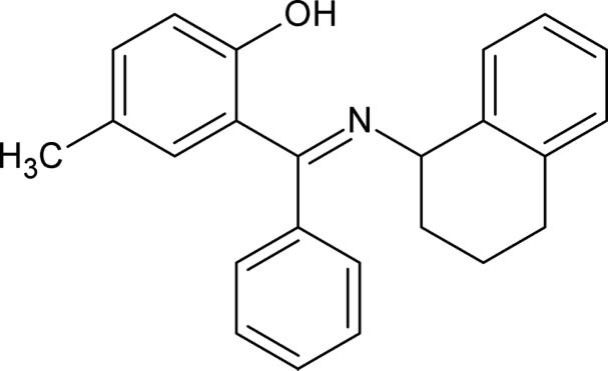


## Experimental

### 

#### Crystal data

C_24_H_23_NO
*M* *_r_* = 341.43
Triclinic, 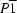

*a* = 10.121 (3) Å
*b* = 10.370 (2) Å
*c* = 10.482 (2) Å
α = 95.181 (3)°
β = 112.830 (3)°
γ = 106.243 (4)°
*V* = 948.7 (4) Å^3^
*Z* = 2
Mo *K*α radiation
μ = 0.07 mm^−1^
*T* = 298 (2) K
0.41 × 0.21 × 0.20 mm

#### Data collection

Bruker SMART CCD area-detector diffractometer
Absorption correction: none
5027 measured reflections
3467 independent reflections
2220 reflections with *I* > 2σ(*I*)
*R*_int_ = 0.015

#### Refinement

*R*[*F* ^2^ > 2σ(*F* ^2^)] = 0.048
*wR*(*F* ^2^) = 0.128
*S* = 1.02
3467 reflections
237 parameters
H-atom parameters constrained
Δρ_max_ = 0.13 e Å^−3^
Δρ_min_ = −0.15 e Å^−3^

### 

Data collection: *SMART* (Bruker, 1997 ▶); cell refinement: *SAINT* (Bruker, 1997 ▶); data reduction: *SAINT*; program(s) used to solve structure: *SHELXS97* (Sheldrick, 2008 ▶); program(s) used to refine structure: *SHELXL97* (Sheldrick, 2008 ▶); molecular graphics: *SHELXTL* (Sheldrick, 2008 ▶); software used to prepare material for publication: *SHELXTL*.

## Supplementary Material

Crystal structure: contains datablocks I, global. DOI: 10.1107/S1600536808030316/is2336sup1.cif

Structure factors: contains datablocks I. DOI: 10.1107/S1600536808030316/is2336Isup2.hkl

Additional supplementary materials:  crystallographic information; 3D view; checkCIF report

## Figures and Tables

**Table 1:** Hydrogen-bond geometry (Å, °)

*D*—H⋯*A*	*D*—H	H⋯*A*	*D*⋯*A*	*D*—H⋯*A*
O1—H1⋯N1	0.82	1.81	2.541 (2)	147

**Fig. 1.**
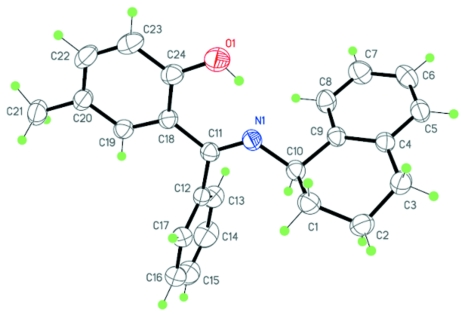
The asymmetric unit of (I), showing the atom-labelling scheme. Displacement ellipsoids are drawn at the 30% probability level and H atoms are shown as spheres of arbitrary radii.

**Fig. 2.**
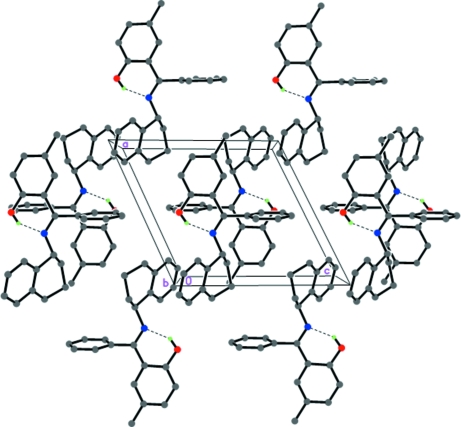
A packing diagram of (I), view down the b axis, showing the O1—H1···N1 hydrogen bonds (dashed lines). H atoms not involved in the hydrogen bonds have been omitted.

## supplementary crystallographic information

### Comment

The synthesis of Schiff bases with a variety of functionalities is an important subject of research because this class of compounds are easily synthesized and have been widely used as ligands in the formation of almost all metal ions and asymmetric reactions (Elmali & Eleman, 1998; Elmali *et al.*, 1998; Cavell *et al.*, 2002; Nakayama *et al.*, 2004; Varlamov *et al.*, 2003; Takenaka *et al.*, 2002; Desimani *et al.*, 1995; Bernaldi *et al.*, 1996; Kureshy *et al.*, 1996; Jacobsen *et al.*, 1997).

In this paper, we report the molecular structure of 4-methyl-2-[(*E*)-phenyl(1,2,3,4-tetrahydronaphthalen-1-ylimino)methyl]\ phenol, (I), which was initially prepared to test its catalytic activity. The Schiff base was prepared by conventional condensation of 1,2,3,4-tetrahydronaphthalen-1-amine with (2-hydroxy-5-methylphenyl)(phenyl)methanone in methanol.

There is an intramolecular O1—H1···N1 hydrogen bond (Table 1). Phenol atom O1 acts as a hydrogen-bond donor to atom N1, with O1··· N1= 2.541 (2) Å, which indicates a comparatively strong intramolecular hydrogen bond. This distance is significantly shorter than the sum (3.07 Å) of the van der Waals radii for N and O atoms. The O1—H1···N1 hydrogen bond in (I) completes a six-membered ring (C11/C18/C24/O1/H1/N1), which increases the stability of this compound. However, no aromatic π-π stacking interactions are present in the structure of (I).

The C12—C17 and C18—C24 aromatic rings are approximately vertical, the dihedral angle between their planes being 81.53 (11)°; the dihedral angle between the planes of the C4—C9 and C12—C17 aromatic rings is 75.35 (12)°, while that between the C4—C9 and C19—C24 planes is 71.1 (10)°.

### Experimental

1,2,3,4-Tetrahydronaphthalen-1-amine (0.9 mmol) and (2-hydroxy-5-methylphenyl)(phenyl)methanone (0.9 mmol) were dissolved in methanol (10 ml) and reacted at room temperature for 48 h. After removal of the solvent, the yellow solid was obtained. Single crystals suitable for X-ray diffraction were grown by slow evaporation from an ethanol solution at room temperature.

### Refinement

All H atoms were included in calculated positions and treated as riding on their parent atoms, with O—H = 0.82 Å, aromatic C—H = 0.93 Å, methyl C—H = 0.96 Å, methylene C—H = 0.97 Å and methine C—H = 0.98 Å, and with *U*_iso_(H) = 1.2*U*_eq_(C) or 1.5*U*_eq_(methyl C, O).

### Crystal data

**Table:**

C_24_H_23_NO	*Z* = 2
*M**_r_* = 341.43	*F*(000) = 364
Triclinic, *P*1	*D*_x_ = 1.195 Mg m^−^^3^
Hall symbol: -P 1	Mo *K*α radiation, λ = 0.71073 Å
*a* = 10.121 (3) Å	Cell parameters from 1182 reflections
*b* = 10.370 (2) Å	θ = 2.3–23.4°
*c* = 10.482 (2) Å	µ = 0.07 mm^−^^1^
α = 95.181 (3)°	*T* = 298 K
β = 112.830 (3)°	Block, yellow
γ = 106.243 (4)°	0.41 × 0.21 × 0.20 mm
*V* = 948.7 (4) Å^3^	

### Data collection

**Table:**

Bruker SMART APEX2 CCD area-detector diffractometer	2220 reflections with *I* > 2σ(*I*)
Radiation source: fine-focus sealed tube	*R*_int_ = 0.015
graphite	θ_max_ = 25.5°, θ_min_ = 2.1°
φ and ω scans	*h* = −11→12
5027 measured reflections	*k* = −9→12
3467 independent reflections	*l* = −12→12

### Refinement

**Table:**

Refinement on *F*^2^	Primary atom site location: structure-invariant direct methods
Least-squares matrix: full	Secondary atom site location: difference Fourier map
*R*[*F*^2^ > 2σ(*F*^2^)] = 0.048	Hydrogen site location: inferred from neighbouring sites
*wR*(*F*^2^) = 0.128	H-atom parameters constrained
*S* = 1.02	*w* = 1/[σ^2^(*F*_o_^2^) + (0.0565*P*)^2^ + 0.046*P*] where *P* = (*F*_o_^2^ + 2*F*_c_^2^)/3
3467 reflections	(Δ/σ)_max_ < 0.001
237 parameters	Δρ_max_ = 0.13 e Å^−^^3^
0 restraints	Δρ_min_ = −0.15 e Å^−^^3^

### Special details

**Table:**

Geometry. All e.s.d.'s (except the e.s.d. in the dihedral angle between two l.s. planes) are estimated using the full covariance matrix. The cell e.s.d.'s are taken into account individually in the estimation of e.s.d.'s in distances, angles and torsion angles; correlations between e.s.d.'s in cell parameters are only used when they are defined by crystal symmetry. An approximate (isotropic) treatment of cell e.s.d.'s is used for estimating e.s.d.'s involving l.s. planes.
Refinement. Refinement of *F*^2^ against ALL reflections. The weighted *R*-factor *wR* and goodness of fit *S* are based on *F*^2^, conventional *R*-factors *R* are based on *F*, with *F* set to zero for negative *F*^2^. The threshold expression of *F*^2^ > σ(*F*^2^) is used only for calculating *R*-factors(gt) *etc*. and is not relevant to the choice of reflections for refinement. *R*-factors based on *F*^2^ are statistically about twice as large as those based on *F*, and *R*- factors based on ALL data will be even larger.

### Fractional atomic coordinates and isotropic or equivalent isotropic displacement parameters (Å^2^)

**Table:**

	*x*	*y*	*z*	*U*_iso_*/*U*_eq_	
C1	0.1262 (2)	0.4914 (2)	0.3438 (2)	0.0635 (5)	
H1A	0.1775	0.4751	0.4372	0.076*	
H1B	0.1420	0.4344	0.2763	0.076*	
C2	−0.0430 (2)	0.4527 (2)	0.3033 (2)	0.0674 (6)	
H2A	−0.0832	0.3591	0.3121	0.081*	
H2B	−0.0595	0.5138	0.3668	0.081*	
C3	−0.1248 (2)	0.4641 (2)	0.1520 (2)	0.0668 (6)	
H3A	−0.1229	0.3917	0.0880	0.080*	
H3B	−0.2307	0.4505	0.1305	0.080*	
C4	−0.0538 (2)	0.60102 (18)	0.12713 (18)	0.0504 (5)	
C5	−0.1381 (2)	0.6473 (2)	0.0128 (2)	0.0628 (5)	
H5	−0.2384	0.5927	−0.0470	0.075*	
C6	−0.0767 (3)	0.7713 (2)	−0.0136 (2)	0.0746 (6)	
H6	−0.1352	0.8004	−0.0905	0.089*	
C7	0.0713 (3)	0.8527 (2)	0.0731 (2)	0.0760 (6)	
H7	0.1138	0.9368	0.0549	0.091*	
C8	0.1561 (2)	0.8091 (2)	0.1869 (2)	0.0643 (5)	
H8	0.2563	0.8648	0.2459	0.077*	
C9	0.0960 (2)	0.68399 (18)	0.21587 (18)	0.0492 (4)	
C10	0.19315 (19)	0.64187 (19)	0.34485 (18)	0.0529 (5)	
H10	0.2032	0.6984	0.4305	0.063*	
C11	0.4687 (2)	0.72766 (17)	0.46336 (18)	0.0485 (4)	
C12	0.47002 (19)	0.76700 (19)	0.60484 (18)	0.0495 (4)	
C13	0.4491 (2)	0.8879 (2)	0.6440 (2)	0.0685 (6)	
H13	0.4349	0.9471	0.5823	0.082*	
C14	0.4492 (3)	0.9210 (3)	0.7741 (3)	0.0938 (8)	
H14	0.4349	1.0024	0.7999	0.113*	
C15	0.4702 (3)	0.8349 (4)	0.8654 (3)	0.0999 (9)	
H15	0.4707	0.8582	0.9534	0.120*	
C16	0.4906 (2)	0.7147 (3)	0.8285 (2)	0.0863 (7)	
H16	0.5039	0.6559	0.8907	0.104*	
C17	0.4912 (2)	0.6809 (2)	0.6982 (2)	0.0656 (6)	
H17	0.5061	0.5995	0.6733	0.079*	
C18	0.61531 (19)	0.75267 (17)	0.45517 (19)	0.0489 (4)	
C19	0.7536 (2)	0.81930 (18)	0.5754 (2)	0.0547 (5)	
H19	0.7509	0.8483	0.6606	0.066*	
C20	0.8942 (2)	0.8440 (2)	0.5731 (2)	0.0609 (5)	
C21	1.0404 (2)	0.9151 (2)	0.7060 (2)	0.0814 (7)	
H21A	1.0535	0.8547	0.7706	0.122*	
H21B	1.0357	0.9978	0.7502	0.122*	
H21C	1.1250	0.9379	0.6816	0.122*	
C22	0.8940 (3)	0.7998 (2)	0.4443 (3)	0.0730 (6)	
H22	0.9868	0.8141	0.4397	0.088*	
C23	0.7608 (3)	0.7353 (2)	0.3232 (3)	0.0747 (6)	
H23	0.7649	0.7068	0.2385	0.090*	
C24	0.6212 (2)	0.7125 (2)	0.3262 (2)	0.0592 (5)	
N1	0.34550 (17)	0.67080 (15)	0.34810 (15)	0.0552 (4)	
O1	0.49367 (17)	0.65077 (17)	0.20388 (15)	0.0813 (5)	
H1	0.4183	0.6450	0.2182	0.122*	

### Atomic displacement parameters (Å^2^)

**Table:**

	*U*^11^	*U*^22^	*U*^33^	*U*^12^	*U*^13^	*U*^23^
C1	0.0662 (13)	0.0706 (14)	0.0607 (13)	0.0295 (11)	0.0287 (11)	0.0228 (10)
C2	0.0667 (14)	0.0671 (13)	0.0752 (14)	0.0204 (10)	0.0374 (11)	0.0244 (11)
C3	0.0538 (12)	0.0704 (14)	0.0669 (14)	0.0124 (10)	0.0241 (10)	0.0094 (10)
C4	0.0490 (11)	0.0586 (12)	0.0431 (10)	0.0193 (9)	0.0203 (9)	0.0052 (8)
C5	0.0535 (12)	0.0779 (15)	0.0499 (12)	0.0257 (11)	0.0147 (10)	0.0070 (10)
C6	0.0799 (16)	0.0851 (17)	0.0579 (13)	0.0412 (14)	0.0188 (12)	0.0217 (12)
C7	0.0871 (17)	0.0626 (14)	0.0782 (15)	0.0278 (12)	0.0320 (14)	0.0266 (12)
C8	0.0586 (12)	0.0538 (13)	0.0659 (13)	0.0134 (10)	0.0171 (10)	0.0102 (10)
C9	0.0486 (11)	0.0530 (11)	0.0436 (10)	0.0189 (9)	0.0179 (8)	0.0053 (8)
C10	0.0490 (11)	0.0632 (13)	0.0457 (10)	0.0216 (9)	0.0189 (9)	0.0081 (9)
C11	0.0509 (11)	0.0511 (11)	0.0477 (11)	0.0221 (8)	0.0218 (9)	0.0132 (8)
C12	0.0383 (10)	0.0594 (12)	0.0466 (10)	0.0142 (8)	0.0166 (8)	0.0084 (9)
C13	0.0674 (14)	0.0650 (14)	0.0663 (14)	0.0160 (10)	0.0296 (11)	0.0015 (10)
C14	0.0892 (18)	0.098 (2)	0.0801 (18)	0.0179 (14)	0.0410 (15)	−0.0227 (15)
C15	0.0812 (18)	0.146 (3)	0.0532 (15)	0.0165 (17)	0.0319 (13)	−0.0062 (17)
C16	0.0619 (15)	0.134 (2)	0.0597 (15)	0.0237 (14)	0.0266 (12)	0.0350 (15)
C17	0.0585 (13)	0.0866 (15)	0.0605 (13)	0.0296 (11)	0.0294 (10)	0.0264 (11)
C18	0.0505 (11)	0.0514 (11)	0.0546 (11)	0.0239 (9)	0.0267 (9)	0.0182 (8)
C19	0.0558 (12)	0.0598 (12)	0.0603 (12)	0.0262 (9)	0.0307 (10)	0.0227 (9)
C20	0.0527 (12)	0.0612 (12)	0.0824 (15)	0.0251 (10)	0.0359 (11)	0.0316 (11)
C21	0.0521 (13)	0.0895 (17)	0.1002 (18)	0.0228 (12)	0.0291 (13)	0.0329 (13)
C22	0.0632 (15)	0.0807 (15)	0.1012 (18)	0.0317 (12)	0.0543 (14)	0.0319 (13)
C23	0.0822 (16)	0.0838 (16)	0.0843 (16)	0.0341 (13)	0.0577 (14)	0.0206 (13)
C24	0.0635 (13)	0.0644 (13)	0.0600 (13)	0.0256 (10)	0.0344 (11)	0.0149 (10)
N1	0.0512 (9)	0.0688 (10)	0.0468 (9)	0.0252 (8)	0.0197 (8)	0.0108 (7)
O1	0.0767 (10)	0.1065 (12)	0.0592 (9)	0.0281 (9)	0.0338 (8)	0.0020 (8)

### Geometric parameters (Å, °)

**Table:**

C1—C10	1.514 (3)	C12—C17	1.379 (2)
C1—C2	1.515 (3)	C12—C13	1.382 (3)
C1—H1A	0.9700	C13—C14	1.375 (3)
C1—H1B	0.9700	C13—H13	0.9300
C2—C3	1.511 (3)	C14—C15	1.365 (4)
C2—H2A	0.9700	C14—H14	0.9300
C2—H2B	0.9700	C15—C16	1.365 (4)
C3—C4	1.498 (3)	C15—H15	0.9300
C3—H3A	0.9700	C16—C17	1.382 (3)
C3—H3B	0.9700	C16—H16	0.9300
C4—C5	1.391 (2)	C17—H17	0.9300
C4—C9	1.392 (2)	C18—C19	1.399 (2)
C5—C6	1.366 (3)	C18—C24	1.407 (3)
C5—H5	0.9300	C19—C20	1.383 (3)
C6—C7	1.371 (3)	C19—H19	0.9300
C6—H6	0.9300	C20—C22	1.385 (3)
C7—C8	1.374 (3)	C20—C21	1.509 (3)
C7—H7	0.9300	C21—H21A	0.9600
C8—C9	1.385 (2)	C21—H21B	0.9600
C8—H8	0.9300	C21—H21C	0.9600
C9—C10	1.517 (2)	C22—C23	1.375 (3)
C10—N1	1.472 (2)	C22—H22	0.9300
C10—H10	0.9800	C23—C24	1.380 (3)
C11—N1	1.286 (2)	C23—H23	0.9300
C11—C18	1.471 (2)	C24—O1	1.351 (2)
C11—C12	1.496 (2)	O1—H1	0.8200
			
C10—C1—C2	110.10 (15)	C17—C12—C13	118.94 (19)
C10—C1—H1A	109.6	C17—C12—C11	119.75 (17)
C2—C1—H1A	109.6	C13—C12—C11	121.31 (17)
C10—C1—H1B	109.6	C14—C13—C12	120.2 (2)
C2—C1—H1B	109.6	C14—C13—H13	119.9
H1A—C1—H1B	108.2	C12—C13—H13	119.9
C3—C2—C1	109.48 (17)	C15—C14—C13	120.2 (2)
C3—C2—H2A	109.8	C15—C14—H14	119.9
C1—C2—H2A	109.8	C13—C14—H14	119.9
C3—C2—H2B	109.8	C14—C15—C16	120.5 (2)
C1—C2—H2B	109.8	C14—C15—H15	119.8
H2A—C2—H2B	108.2	C16—C15—H15	119.8
C4—C3—C2	112.22 (16)	C15—C16—C17	119.6 (2)
C4—C3—H3A	109.2	C15—C16—H16	120.2
C2—C3—H3A	109.2	C17—C16—H16	120.2
C4—C3—H3B	109.2	C12—C17—C16	120.5 (2)
C2—C3—H3B	109.2	C12—C17—H17	119.8
H3A—C3—H3B	107.9	C16—C17—H17	119.8
C5—C4—C9	118.63 (18)	C19—C18—C24	117.86 (17)
C5—C4—C3	119.88 (18)	C19—C18—C11	120.89 (16)
C9—C4—C3	121.49 (16)	C24—C18—C11	121.24 (16)
C6—C5—C4	121.44 (19)	C20—C19—C18	122.96 (18)
C6—C5—H5	119.3	C20—C19—H19	118.5
C4—C5—H5	119.3	C18—C19—H19	118.5
C5—C6—C7	120.02 (19)	C19—C20—C22	117.00 (19)
C5—C6—H6	120.0	C19—C20—C21	121.0 (2)
C7—C6—H6	120.0	C22—C20—C21	121.95 (19)
C6—C7—C8	119.4 (2)	C20—C21—H21A	109.5
C6—C7—H7	120.3	C20—C21—H21B	109.5
C8—C7—H7	120.3	H21A—C21—H21B	109.5
C7—C8—C9	121.49 (19)	C20—C21—H21C	109.5
C7—C8—H8	119.3	H21A—C21—H21C	109.5
C9—C8—H8	119.3	H21B—C21—H21C	109.5
C8—C9—C4	118.98 (17)	C23—C22—C20	121.99 (19)
C8—C9—C10	119.51 (16)	C23—C22—H22	119.0
C4—C9—C10	121.50 (17)	C20—C22—H22	119.0
N1—C10—C1	110.00 (14)	C22—C23—C24	120.6 (2)
N1—C10—C9	107.72 (15)	C22—C23—H23	119.7
C1—C10—C9	112.97 (15)	C24—C23—H23	119.7
N1—C10—H10	108.7	O1—C24—C23	118.30 (19)
C1—C10—H10	108.7	O1—C24—C18	122.10 (17)
C9—C10—H10	108.7	C23—C24—C18	119.60 (19)
N1—C11—C18	117.95 (16)	C11—N1—C10	122.17 (15)
N1—C11—C12	123.35 (16)	C24—O1—H1	109.5
C18—C11—C12	118.69 (15)		
			
C10—C1—C2—C3	65.2 (2)	C12—C13—C14—C15	0.1 (3)
C1—C2—C3—C4	−51.8 (2)	C13—C14—C15—C16	−0.4 (4)
C2—C3—C4—C5	−159.33 (17)	C14—C15—C16—C17	0.6 (4)
C2—C3—C4—C9	20.9 (3)	C13—C12—C17—C16	0.4 (3)
C9—C4—C5—C6	0.2 (3)	C11—C12—C17—C16	−179.06 (17)
C3—C4—C5—C6	−179.57 (18)	C15—C16—C17—C12	−0.6 (3)
C4—C5—C6—C7	0.3 (3)	N1—C11—C18—C19	177.33 (16)
C5—C6—C7—C8	−0.6 (3)	C12—C11—C18—C19	−3.8 (2)
C6—C7—C8—C9	0.4 (3)	N1—C11—C18—C24	−1.7 (2)
C7—C8—C9—C4	0.0 (3)	C12—C11—C18—C24	177.24 (16)
C7—C8—C9—C10	−178.60 (18)	C24—C18—C19—C20	−1.6 (3)
C5—C4—C9—C8	−0.3 (3)	C11—C18—C19—C20	179.42 (15)
C3—C4—C9—C8	179.40 (17)	C18—C19—C20—C22	0.2 (3)
C5—C4—C9—C10	178.29 (16)	C18—C19—C20—C21	−179.38 (17)
C3—C4—C9—C10	−2.0 (3)	C19—C20—C22—C23	0.6 (3)
C2—C1—C10—N1	−165.74 (15)	C21—C20—C22—C23	−179.87 (19)
C2—C1—C10—C9	−45.3 (2)	C20—C22—C23—C24	0.1 (3)
C8—C9—C10—N1	−45.2 (2)	C22—C23—C24—O1	178.91 (19)
C4—C9—C10—N1	136.15 (17)	C22—C23—C24—C18	−1.5 (3)
C8—C9—C10—C1	−166.94 (16)	C19—C18—C24—O1	−178.26 (16)
C4—C9—C10—C1	14.5 (2)	C11—C18—C24—O1	0.8 (3)
N1—C11—C12—C17	98.4 (2)	C19—C18—C24—C23	2.2 (3)
C18—C11—C12—C17	−80.5 (2)	C11—C18—C24—C23	−178.77 (16)
N1—C11—C12—C13	−81.1 (2)	C18—C11—N1—C10	−179.77 (14)
C18—C11—C12—C13	100.0 (2)	C12—C11—N1—C10	1.4 (3)
C17—C12—C13—C14	−0.2 (3)	C1—C10—N1—C11	−100.19 (19)
C11—C12—C13—C14	179.30 (18)	C9—C10—N1—C11	136.28 (16)

### Hydrogen-bond geometry (Å, °)

**Table:**

*D*—H···*A*	*D*—H	H···*A*	*D*···*A*	*D*—H···*A*
O1—H1···N1	0.82	1.81	2.541 (2)	147
